# *PIM1* and *CD79B* Mutation Status Impacts the Outcome of Primary Diffuse Large B-Cell Lymphoma of the CNS

**DOI:** 10.3389/fonc.2022.824632

**Published:** 2022-02-09

**Authors:** Jihao Zhou, Min Zuo, Lifeng Li, Fang Li, Peng Ke, Yangying Zhou, Yaping Xu, Xuan Gao, Yanfang Guan, Xuefeng Xia, Xin Yi, Xinyou Zhang, Yuhua Huang

**Affiliations:** ^1^ Department of Hematology, Shenzhen People’s Hospital (The Second Clinical Medical College, Jinan University, The First Affiliated Hospital, Southern University of Science and Technology), Shenzhen, China; ^2^ Department of Pathology, Shenzhen People’s Hospital (The Second Clinical Medical College, Jinan University, The First Affiliated Hospital, Southern University of Science and Technology), Shenzhen, China; ^3^ Medical Center, Geneplus-Beijing, Beijing, China; ^4^ Geneplus-Beijing Institute, Beijing, China; ^5^ Department of Oncology, Xiangya Hospital, Central South University, Changsha, China; ^6^ Sun Yat-sen University Cancer Center, State Key Laboratory of Oncology in South China, Collaborative Innovation Center for Cancer Medicine, Guangzhou, China; ^7^ Department of Pathology, Sun Yat-sen University Cancer Center, Guangzhou, China

**Keywords:** central nervous system, diffuse large B-cell lymphoma, molecular classification, *PIM1*, *CD79B*

## Abstract

Primary diffuse large B cell lymphoma of the central nervous system (CNS DLBCL) is a rare malignancy with a distinct genetic profile. The clinicopathological significance of the mutation patterns remains unknown. Forty cases of primary CNS DLBCL were subjected to targeted exome sequencing covering 413 genes, including *MYD88*, *CD79B* and *PIM1*. Mutational analysis recognized two groups. The CDP (including *CD79B* and/or *PIM1*mutations) group was identified in 27 cases (67.5%), and the non-CDP (without *CD79B* and PIM1 mutations) group was identified in 13 cases 32.5%). The CDP group tended to occur in older patients (median age 57.0 vs. 48.4 years, *p*=0.015). Patients in the CDP group had a significantly longer 2-year overall survival (OS) (76% and 40%, *p*=0.0372) than those in the non-CDP group. Multivariate analysis revealed that age less than 60 years, no MYC and BCL2 double expression, and CDP group were three independent risk factors indicating favorable OS. PyClone analysis revealed the subcloning heterogeneity between the groups. In addition, transcriptional sequencing was successfully performed in 8 cases. A total of 131 genes were significantly differentially expressed between these two groups. The major categories of biological processes that were significantly altered between these two groups related to intracellular metabolism mechanisms. We developed a new molecular classification to divide CNS DLBCL into CDP and non-CDP groups based on *CD79B* and *PIM1* mutational status. Patients with *PIM1* and/or *CD79B* mutations had favorable long-term survival after high-dose methotrexate-based polychemotherapy.

## Introduction

Primary central nervous system (CNS) diffuse large B-cell lymphoma (DLBCL) is a rare malignancy that only accounts for <1% of all non-Hodgkin lymphomas (NHL) and 2.4–3% of all brain tumors. In recent decades, an increased incidence of CNS DLBCL has been reported among patients aged >60 years (1). Currently, the treatment of CNS DLBCL is primarily based on high-dose methotrexate (HD-MTX), followed by whole-brain radiation (WBR) and/or autologous hematopoietic stem cell transplantation (ASCT) (2). Patients with CNS DLBCL have a remarkably worse outcome than patients with systemic DLBCL.

The extreme genetic and phenotypic heterogeneity of DLBCL presents a great challenge to the development of precision therapies. Since 2000, gene expression profiling has first subclassified DLBCL into the geminal-center B cell type (GCB), activated B cell type (ABC) and unclassified type (UC). This “cell-of-origin” (COO) methodology showed great prognostic significance and dramatically changed our clinical practice (3). However, the majority of primary CNS DLBCL belongs to the non-GCB subtype, which limits the use of COO methodology by immunohistochemistry of systemic DLBCL to CNS DLBCL to predict patient outcome.

With the development of next-generation sequencing (NGS), the molecular classification of DLBCL has developed from expression profiling to genetic profiling. Genetic subtypes of DLBCL show enormous potential in predicting prognosis and guiding precision therapy (4–6). Systemic DLBCL can be subdivided into seven genetic subtypes, including MCD (including *MYD88* ^L265P^ and *CD79B* mutations), N1 (including NOTCH1 mutations), A53 (characterized by *TP53* mutations and deletions), BN2 (including *BCL6* translocations and *NOTCH2* mutations), ST2 (with recurrent *SGK1* and *TET2* mutations), *EZB Myc*+ and *EZB Myc*- (4). CNS DLBCLs frequently harbored *MYD88*, *CD79B* and/or *PIM1* mutations. According to the classification, approximately 37% of CNS DLBCL cases fall into the so-called MCD subtype (7, 8). Unfortunately, the molecular classification of systemic DLBCL is not applicable to CNS DLBCL to predict patient outcome. Therefore, a new molecular classification is needed to elucidate the relationship between the mutation patterns and clinicopathological features, as well as the patients’ outcomes.

In present study, we attempted to classify primary CNS DLBCLs into two biologically relevant subgroups based on the mutational status of *CD79B* and *PIM1* by targeted exome sequencing covering 413 genes. Patients harboring *CD79B* and/or *PIM1* mutations belonged to the so-called CDP group, while those without *CD79B* or *PIMI* mutations belonged to the so-called non-CDP group. We analyzed the clinical characteristics and prognosis between the two molecular subgroups. In addition, we performed transcriptional sequencing to find out significantly differentially expressed genes between these two molecular subgroups.

## Materials and Methods

### Patients and Clinicopathological Data

A retrospective cohort of forty patients with primary CNS DLBCL at Shenzhen People’s Hospital and Sun Yat-sen University Cancer Center from January 2016 to October 2019 was enrolled. The exclusion criteria were lymphomas of the dura, intravascular large B-cell lymphoma, lymphomas with evidence of systemic disease or secondary lymphoma, and all immunodeficiency-associated lymphomas. All patients were of Chinese origin and received HD-MTX-based polychemotherapy. Most patients received rituximab treatment at the same time. This study was approved by the ethical committee at Shenzhen People’s Hospital (Reference number: LL-KY-2020199). All patients provided written informed consent for the collection and publication of their medical information. The authenticity of this article has been validated by uploading the key raw data onto the Research Data Deposit public platform (www.researchdata.org.cn), with the approval RDD number as RDDA2021002020. The clinical characteristics of all patients in the present cohort are summarized in **Table 1**.

**Table 1:** Patients’ clinical characteristics between CDP and non-CDP groups.

Characteristics	No. of patients (%)		*P* value
	CDP group (n = 28)	Non-CDP group (n = 12)	
Age			
Mean ± SD	57.04 ± 10.58	48.42 ± 7.40	**0.015**
Gender			
Male	14/28 (50.0%)	8/12 (66.7%)	0.491
Female	14/28 (50.0%)	4/12 (33.3%)	
Subtypes by IHC			
GCB	6/28 (21.4%)	5/12 (41.7%)	0.254
Non-GCB	22/28 (78.6%)	7/12 (58.3%)	
No of Lesions			
Single	17/28 (60.7%)	7/12 (58.3%)	1.000
Multiple	11/28 (39.3%)	5/12 (41.7%)	
MYC/BCL2 Double Expression			
Yes	10/20 (50.0%)	5/11 (45.5%)	1.000
No	10/20 (50.0%)	6/11 (54.5%)	
MCD subtypes			
Yes	12/28 (42.9%)	0/12 (0.00%)	**0.007**
No	16/28 (57.1%)	12/12 (100%)	
Whole Brain Radiation			
Yes	16/28 (57.1%)	5/12 (41.7%)	0.369
No	12/28 (42.9%)	7/12 (58.3%)	
Tumor mutation burden (TMB)			
Mean ± SD	11.6 ± 3.3	8.7 ± 5.0	**0.019**

GCB, germinal center B cell typle; MCD, MYD88 and CD79B double mutation.

The bold values indicate significant differences.

### Immunohistochemical (IHC) Staining and EBV-Encoded RNA (EBER) *In Situ* Hybridization (ISH)

The specimens of these forty cases were formalin fixed and paraffin embedded (FFPE) and then sectioned at 4.0 mm thickness. The sections were stained using hematoxylin and eosin staining and were used for IHC and ISH examination. IHC was performed to analyze the expression of CD20, CD3, CD10, BCL6, MUM1, MYC, BCL2 and Ki67 in all cases using a BenchMark ULTRA automatic immunostaining device according to the manufacturer’s instructions. The EBV Probe *In Situ* Hybridization Kit (ISH-6021, Zhongshan Golden Bridge Biotechnology Co. Ltd., Beijing, China) was used to detect EBERs according to the manufacturer’s protocol. The Hans algorithm was used to classify the COO subtypes of CNS DLBCL

### Targeted Exome Next-Generation Sequencing

The FFPE tissue samples isolated from forty CNS DLBCL patients were sequenced using commercial DNA sequencing services (GenePlus Co. Beijing, China). Genomic DNA was isolated from FFPE tumor samples using the QIAamp DNA FFPE Tissue kit (Qiagen GmbH, Hilden, Germany). For library preparation, tumor DNA was sheared into 200–250-bp fragments using a Covaris S2 instrument (Woburn, MA, USA), and indexed NGS libraries were prepared using the DNA Library Preparation Kit for MGISeq-2000 (BGI, Shenzhen, China). All libraries were hybridized to custom-designed biotinylated oligonucleotide probes (IDT, Coralville, IA, USA) covering 413 genes (**Table S1**). DNA sequencing was performed using the MGISeq-2000 Sequencing System (BGI, Shenzhen, China) per the manufacturer’s guidelines, which generated 3 Gb of data from tumor DNA. Additional detailed information regarding target region captures, NGS and somatic mutation calling of tumor DNA were as previously described (9, 10). The raw sequence data reported in this paper have been deposited in the Genome Sequence Archive of the BIG Data Center at the Beijing Institute of Genomics, Chinese Academy of Science, under the accession number HRA001659 (http://bigd.big.ac.cn/gsa-human). Code is available from corresponding author on reasonable request.

### PyClone Analysis

PyClone was used to analyze the clonal population structure of tumor samples from each patient (11). The copy number information of each single nucleotide variation (SNV) was used as input. Variants located in the cluster with the greatest mean cancer cell fraction (CCF) were defined as clonal, and the rest were subclonal.

### RNA Sequencing and Differential Gene Expression Analysis

The techniques of performing transcriptional sequencing on FFPE tumor tissue samples were used following the “K. TotalRNA” protocol (12). In brief, we used an RNase H-based method to deplete rRNA from purified total FFPE RNA, followed by library preparation. The libraries were sequenced on BGISeq500 to generate at least 65 million (mean=81 million, sd=6 million) paired-end raw reads with a length of 100 bp for each sample. The computational analysis of RNA-seq data was performed as previously described (12, 13). Briefly, the sequencing reads that passed quality checking were mapped to the human genome reference (hg19) using HISAT2 (14) (version 2.1.0, default setting). FeatureCounts (15) was used to compute the read counts. For differential gene expression analysis between different groups, the original gene expression data were normalized by TMM (the trimmed mean of M values) (16). Only genes with a mean of greater than 15 reads and nonzero values across all samples were retained for normalization, which resulted in a total of 17,375 genes for downstream analysis. The normalized counts were fit into a negative binomial GLM for differential testing using edgeR (17), with tagwise dispersion and risk stratification as the single factor. Multiple testing was corrected by the Benjamini-Hochberg procedure (18) to control the false discovery rate (FDR) and to obtain the adjusted p values.

Gene Ontology (GO) enrichment analysis was performed by GOseq (19), where the differentially expressed genes identified as described above were supplied as the input for genes of interest. ClusterProfiler (20) was also used as a comparison. Gene set enrichment analysis was performed according to Subramanian et al. (21).

### Statistical Analysis

The χ2 test or Fisher’s exact test was used to compare categorical variables, and the nonparametric test was used for continuous variables. The Kaplan–Meier method with the log-rank test was used to calculate the probability of PFS and OS. The effect of risk factors on PFS and OS was evaluated by the Cox proportional hazards regression model. Statistical analysis was performed using the IBM Statistical Package for Social Sciences (SPSS) version 21.0. A P value less than 0.05 was considered significant.

## Results

### Clinicopathological Characteristic

**Figure 1:**
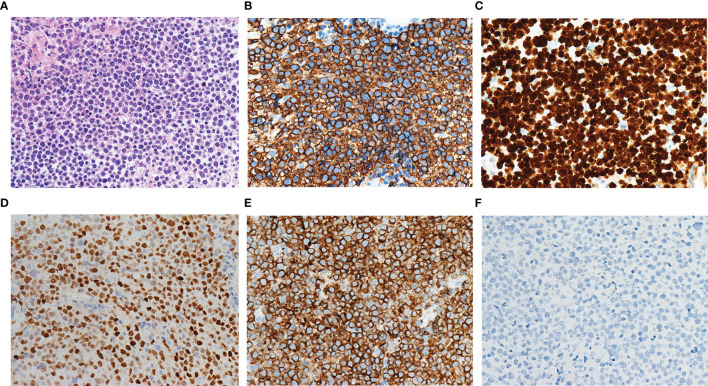
Morphology and immunophenotype of primary CNS DLBCL. The tumor consisted of atypical cells with medium-sized to large round, oval, irregular, or polymorphic nuclei and distinct nucleoli **(A)**. The tumor cells expressed CD20 **(B)** and had a Ki67 proliferation fraction above 90% **(C)**. MYC **(D)** and BCL2 **(E)** double expression were found. All cases were negative for EBER ISH **(F)**.

The age of patients with primary CNS DLBCL in the present cohort ranged from 32 to 80 years, with a median age of 56 years and a male-to-female ratio of 1.1:1. All patients were Chinese. Twenty-four patients (24/40, 60.0%) harbored a solitary tumor, while sixteen patients harbored multiple tumors (16/40, 40.0%). All forty cases showed diffuse and/or perivascular infiltration patterns. Cytomorphologically, the tumors consisted of atypical cells with medium-sized to large round, oval, irregular, or polymorphic nuclei and distinct nucleoli, corresponding to centroblasts or immunoblasts (**Figure 1A**). Twenty-nine cases (29/40, 72.5%) belonged to the non-GCB subtype according to Han’s classification, while eleven cases (11/40, 27.5%) belonged to the GCB subtype. All cases expressed CD20 **(** **Figure 1B** **)**, and most cases (85.0%, 34/40) had a Ki67 proliferation fraction >70% (**Figure 1C**). MYC and BCL2 double expression was found in 50% (15/30) of available cases **(** **Figures 1D, E** **)**. All cases were negative for EBER ISH **(** **Figure 1F** **)**. No MYC/BCL2/BCL6 double/triple hit was found in any cases. Among these 40 patients, 39 patients were successfully followed up, with a median follow-up period of 445 days (ranged 37 to 1354 days).

### Mutational Pattern of Primary CNS DLBCL

All cases of primary CNS DLBCL harbored at least one somatic mutated gene. As shown in **Figure S1A**, *MYD88* (58.5%), *CD79B* (51.2%) and *PIM1* (41.4%) were the three most frequently mutated genes **(** **Figure S1B**). Compared to the TCGA database, primary CNS DLBCL showed a mutation pattern distinct from that of DLBCL-NOS. In particular, primary CNS DLBCL had significantly more *MYD88* mutations and fewer *CREBBP* mutations (**Figures S1C, D**), which was consistent with previously published data (4, 22–26). By univariate survival analysis, *CD79B* mutation correlated with better PFS but showed no benefit to OS, while PIM1 mutation and MYD88 mutation alone showed no influence on either PFS or OS (**Figure S2**).

### Molecular Classification of Primary CNS DLBCL Based on *PIM1* and *CD79B* Mutation Status

**Figure 2:**
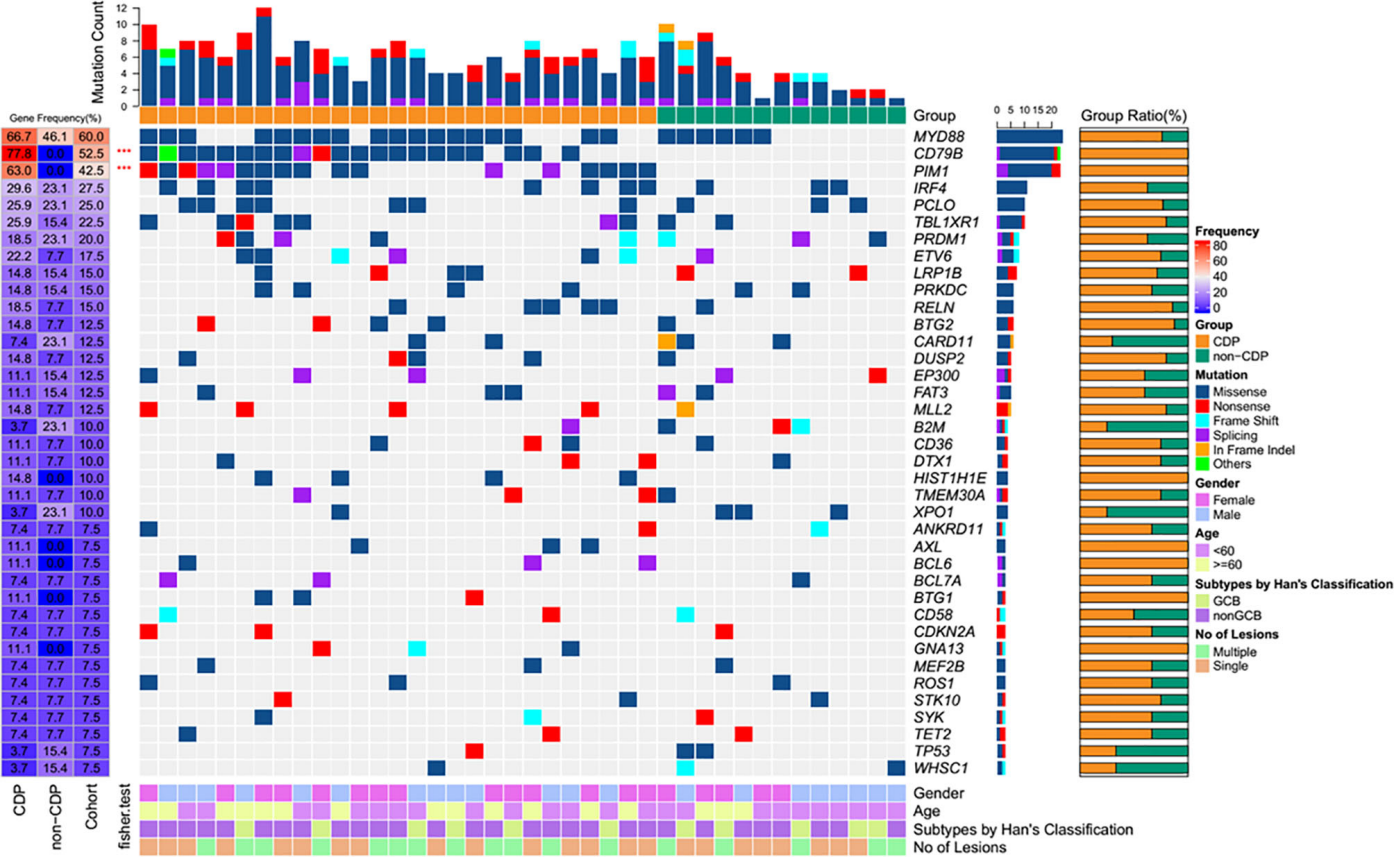
Heatmap of frequently mutated genes and clinical characteristics of the CDP and non-CDP groups.

**Figure 3:**
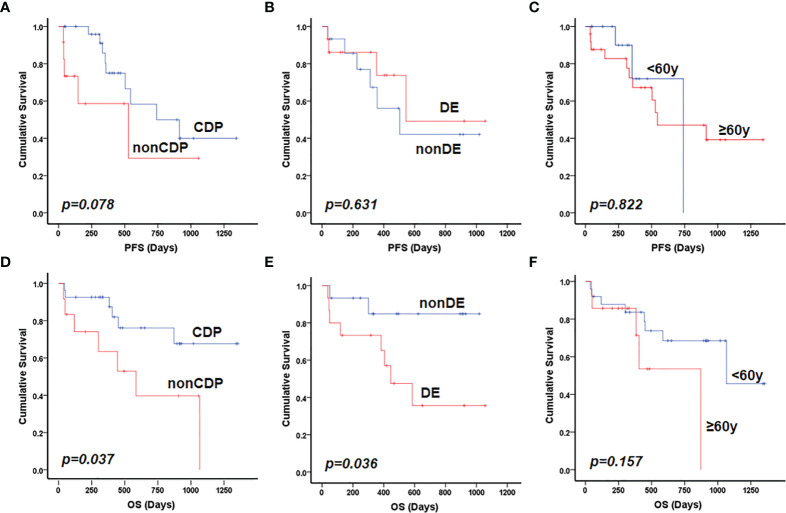
Prognostic factors for patients with primary CNS DLBCL. Patients in the CDP group had longer PFS compared with those in non-CDP group with borderline statistical significance (*p*=0.078) **(A)**; MYC and BCL2 double expression **(B)** and older patient age (>60 years) **(C)** were not significantly related to inferior PFS. Both non-CDP group **(D)** and BCL2 and MYC double expression **(E)** but not older patient age (>60 years) **(F)** were significantly associated with inferior OS in the present cohort by univariate analysis. DE referred to double expression of MYC and BCL2 proteins.

To further explore the clinicopathological significance of the mutated genes in primary CNS DLBCL, we performed hierarchical cluster analysis. Mutational analysis recognized two groups. Group 1, the so-called CDP (with *CD79B* and/or *PIM1* mutation) group, was identified in 27 cases (67.5%). Group 2, the so-called non-CDP (without *CD79B* or *PIM1* mutation) group, was found in 13 cases (32.5%). The CDP group tended to be observed in older patients (median age 57.0 vs. 48.4 y, *p*=0.015) and had significantly higher tumor mutation burden (TMB) (11.6 ± 3.3 vs 8.7 ± 5.0, *p* = 0.019) **(** **Table 1** **)**. The mutated profiles and main clinicopathological characteristics of these two groups are shown in **Figure 2**. Interestingly, in the survival analysis, patients in the CDP group had significantly longer 2-year OS (76% and 40%, *p*=0.037) but not significantly longer 2-year PFS (50.0% and 29%, *p*=0.078) than those in the non-CDP group (**Figure 3** **)**. Multivariate analysis revealed that age less than 60, no MYC and BCL2 double expression by IHC, and CDP group by NGS were three independent risk factors indicating favorable OS **(** **Table 2** **).**

**Table 2:** Univariate and Multivariate analysis of risk factors associated with patients’ survival.

Variations	OS			
	Univariate		Multivariate	
	P value	P value	Exp	95% CI
Double expression by IHC: Yes vs No	**0.036**	**0.044**	6.111	1.051-35.538
CDP vs nonCDP	**0.037**	**0.025**	16.746	1.430-196.090
Age: <60 vs ≥60	0.157	**0.036**	12.937	1.189-140.747
GCB vs nonGCB	0.736	0.234	0.374	0.074-1.887
MCD vs nonMCD	0.465	0.784	0.732	0.079-6.804

IHC, immunohistochemistry; CDP, CD79B and/or PIM1 mutation; GCB, germinal center B cell type; MCD, MYD88 and CD79B double mutation.

The bold values indicate significant differences.

**Figure 4:**
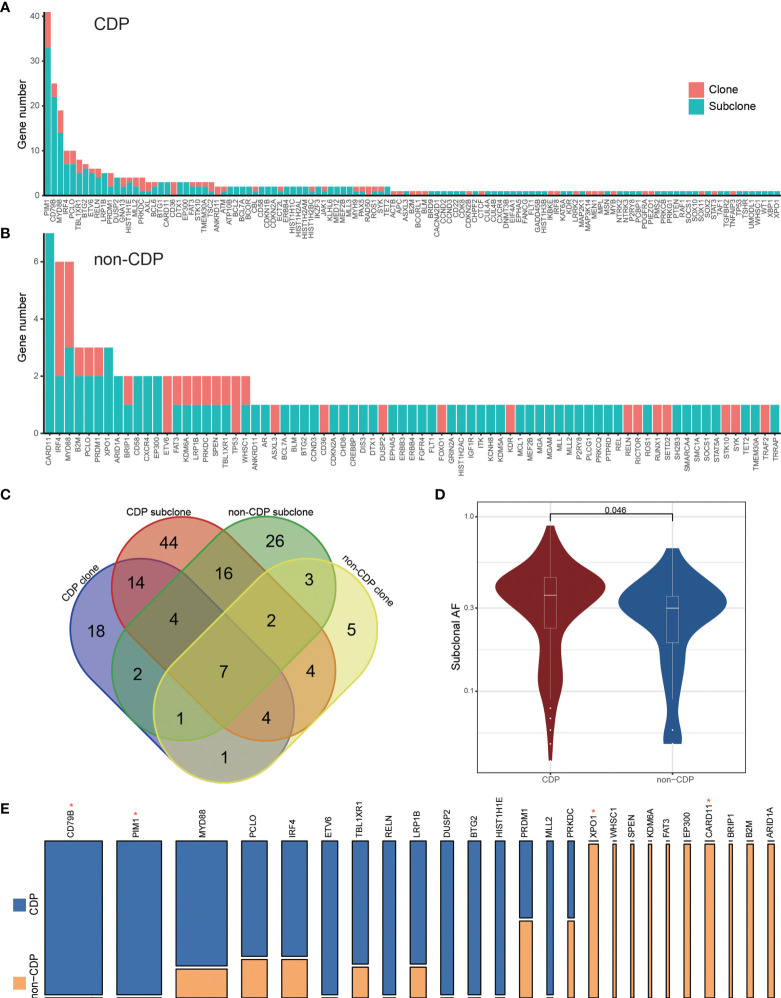
PyClone analysis to CDP and non-CDP groups. The clone and subclonal distribution of mutant genes in the CDP group **(A)** and non-CDP group **(B)**. **(C)** The Venn diagram shows the shared genes status between groups. **(D)** shows the significant difference of allele frequencies in high-frequency subcloned mutant genes between the high CDP and non-CDP groups. **(E)** specifically displays the proportion of high-frequency subcloned mutant genes between the CDP group and non-CDP group. The width of the column represents the number of patients carrying the mutant gene, and the height represents the mutant gene proportion of the two groups. * represents Fisher's exact test, P value < 0.05.

PyClone analysis revealed that there were fewer main clones of the most frequently mutated genes in the non-CDP group than in the CDP group. Among them, 83 main clonal and 230 subclonal gene sites were found in the CDP group (**Figure 4A**), while 34 main clonal and 79 subclonal gene sites were found in the non-CDP group (**Figure 4B**). We found that clonal and subclonal genes were less shared between the CDP group and non-CDP group (**Figure 4C**). Distribution of high-frequency genes in CDP group and non-CDP group was shown in **Figure S3**. Spectrum display of high frequency mutation genes in CDP group and non-CDP group were shown in **Figures S3A, B**. The allele frequency distribution of respectively clonal and subclonal genes in the CDP group and non-CDP were shown in **Figure S3C** and **Figure S3D**. We define the frequency of high-frequency mutation genes as not less than 15% for guaranteed that there are at least two mutations in the non-CDP group and observed that the CDP group and non-CDP group have significant differences in these high-frequency subclonal genes (**Figures 4D, E** **)**, indicating the subcloning heterogeneity between the groups.

### Differential Risk-Related Enrichment of Gene Sets in the CDP and Non-CDP Groups

**Figure 5:**
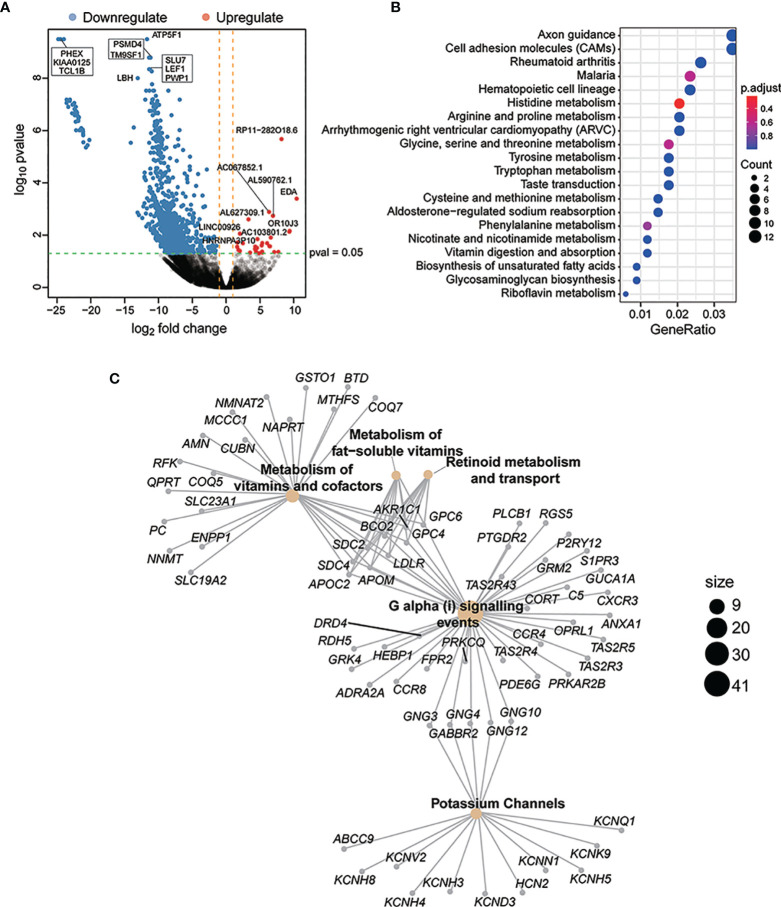
Differential risk-related enrichment of gene sets in CDP and non-CDP groups. A total of 131 genes were identified as significantly differentially expressed with a p value ≤0.05 and |log2F C|≥1 **(A)**. GO enrichment analysis of these genes suggested that some major categories of biological processes, especially for intracellular metabolism mechanism, had been significantly altered for the CDP group compared to the non-CDP group. The y-axis represented different gene function entries, and the x-axis represented the proportion of differentially expressed genes in the corresponding entries to all genes in the entry. The “count”of the circle represented the number of differentially expressed genes enriched in the corresponding entry **(B)**. Reactome network revealed differential gene expression between CDP and non-CDP groups. The yellow dots indicated significantly enriched Reactome entries, the “size” of the dots indicated the number of genes, and the gray dots indicated genes **(C)**.

To investigate transcriptional aberrations between the CDP and non-CDP groups, RNA sequencing analysis was successfully conducted in 8 samples (including 2 samples in the CDP group and 6 samples in the non-CDP group) (**Table S1**). A total of 131 genes were identified as significantly differentially expressed with a p value ≤0.05 and |log2FC| ≥1 (**Figure 5A** **)**. GO enrichment analysis of these genes suggested that some major categories of biological processes, especially intracellular metabolic mechanisms, were significantly altered in the CDP group compared to the non-CDP group (**Figures 5B, C** **)**.

## Discussion

Recently, the classification of DLBCL has promoted the clinical application of precision therapy in systemic DLBCL. However, as an independent rare subtype of DLBCL, due to the following potential reasons, current classification of systemic DLBCL is not applicable to CNS DLBCL: first, most CNS DLBCLs belong to non-GCB subtype based on IHC classification; second, most CNS DLBCL cases harbor *MYD88* and *CD79B* mutations, resulting in a higher frequency of the MCD subtype of CNS DLBCL than systemic DLBCL; and third, the molecular classification of systemic DLBCL originates from patients who receive R-CHOP-like chemoimmunotherapy (R-CHOP is the abbreviated name for the combination of drugs that are commonly used as chemotherapy for DLBCL, including rituximab, cyclophosphamide, doxorubicin, vincristine and prednisone), while the standard treatment of CNS DLBCL is HD-MTX-based chemoimmunotherapy, which has different antitumor mechanisms. To date, molecular classification specific to CNS DLBCL is lacking. Thus, a new molecular classification is needed to elucidate the relationship between the mutation patterns and clinicopathological features and to be used for risk stratification and prognostic prediction.

In our study, we used NGS technology to perform targeted deep sequencing on the coding regions of 413 lymphoma-related genes. The mutation frequency of *MYD88* was the highest, followed by *CD79B*, *PIM1* and *IRF4*, which was consistent with previous studies (4, 22–26). According to previous studies, more than 60% of systemic DLBCL patients can be cured with R-CHOP chemotherapy (27–29). However, patients with CNS DLBCL have a remarkably worse outcome (30–33). The International Extranodal Lymphoma Study Group identified five clinical variables that are correlated with prognoses of CNS DLBCL: age >60 years, elevated lactate dehydrogenase level, Eastern Cooperative Oncology Group (ECOG) performance status >1, high cerebrospinal fluid protein concentration, and location of the tumor in deep brain regions (34). Researchers from Memorial Sloan Kettering Cancer Center (MSKCC) also reported another prognostic score for CNS DLBCL in 2006, which included age more than 50 years and KPS score more than 70 as two independent risk factors indicating unfavorable survival (35). Our present study once again supports the result that age >60 years is an unfavorable risk factor for CNS DLBCL by multivariate analysis. It is worth noting that both the IELSG score and the MSKCC score were developed in pre-rituximab era, patients enrolled received HD-MTX based chemotherapy and/or radiotherapy. Few patients received rituximab in those two studies. However, in our study, most patients enrolled received rituximab in combination with HD-MTX. As was shown in IESLG32 trial, application of rituximab significantly improved survival of CNS DLBCL, and IELSG score did not show any prognostic value when rituximab was used (36). So we believed that new prognostic model is needed to more accurately predict CNS PCNSL’s prognosis in rituximab era. Moreover, we found that MYC and BCL2 double expression by IHC significantly related to unfavorable OS. Besides, the prognostic significance of *CD79B* mutation in primary DLBCL of the CNS remain controversial. Some researchers concluded that *CD79B* mutation predicts better PFS, while others reported that *CD79B* mutation is an unfavorable risk factor for predicting PFS (37, 38). However, cohorts of these studies were relatively small due to the rarity of this disease; and the discrepancy may due to random sampling error. In present study, we first reported that *PIM1* and *CD79B* mutation status impacts the outcome of primary CNS DLBCL after high-dose methotrexate-based polychemotherapy. CNS DLBCL patients without *PIM1* and *CD79B* mutations had inferior long-term survival after HD-MTX-based chemoimmunotherapy in the present study, even though they were younger and had a lower MCD subtype. Therefore, in terms of application, our data suggest that a new molecular classification based on the mutational status of *CD79B* and *PIM1* could be used to predict patient outcomes in CNS DLBCL. What’s more, we found that CDP group had higher TMB than non-CDP group. Since PD1/PDL1-based immunotherapy showed promising efficacy in part of CNS DLBCL (39), it is possible that CDP group may be more sensitive to immune checkpoint inhibitor treatment than non-CDP group, which deserve further exploration.

Although we established such a molecular classification for CNS DLBCL in present study, the underlying mechanism by which *CD79B* and *PIM1* mutational status impact the outcome of primary CNS DLBCL after HD-MTX-based polychemotherapy remains unknown. We tried to make some explanations. On one hand, the PyClone analysis showed that the CDP and non-CDP groups had different subcloning heterogeneity. As was proven in other cancers, such as lung cancer, MDS, or rectal cancer, intratumor heterogeneity is a common phenomenon, representing the evolutionary process of tumor cells (40–42). The clonal hierarchy has a distinct ranking, and the resultant invariant combinations of main clone and subclone mutations yield the specific clinical phenotype and treatment response. On the other hand, RNA sequencing revealed that the altered genes between the two groups primarily enriched in pathways related to intracellular metabolism mechanisms. Metabolic mechanisms are one main factor influencing treatment sensitivity. For example, P-glycoprotein, which is a transmembrane efflux pump, can promote drug efflux in cancer cells, thus reducing the drug concentration in cancer cells and inducing multidrug resistance (43). Another example is that the intergenic single-nucleotide polymorphism of *DHFR* and *FPGS* could affect the levels of MTX in the serum, which results in inadequate treatment intensity and disease relapse after HD-MTX treatment in acute lymphoblastic leukemia patients (44). All patients included in the present cohort received HD-MTX-based chemoimmunotherapy, which is coincidentally an anti-metabolic treatment. Different intracellular metabolic mechanisms may induce different sensitivities to HD-MTX-based treatment.

This study is limited in some ways. Although a relatively large cohort of CNS DLBCL was included, the number of cases was still small due to the rarity of this disease, and some of the results may require verification. Some highly mutated genes in primary CNS DLBCL, such as MUC16, ODZ4 and SLIT2 reported in the previous studies were not covered in our present NGS panel (26, 45). In addition, all patients in the present cohort solely received HD-MTX-based polychemotherapy, while no patient received BTK inhibitors, programmed death 1 (PD-1) and/or thiotepa. Therefore, our molecular classification may only be applicable to patients who receive similar treatments. Furthermore, although we have tried to explain the underlying reasons why this molecular classification impacts patient outcomes based on PyClone analysis and limited cases of RNA sequencing, the mechanism is still unknown.

In conclusion, we developed a new molecular classification to divide CNS DLBCL into CDP and non-CDP groups based on the mutational status of *CD79B* and *PIM1*. CNS DLBCL patients with *PIM1* and/or *CD79B* mutation had favorable long-term survival after HD-MTX-based chemoimmunotherapy. The potential molecular mechanism awaits further investigation.

## Data Availability Statement

The data presented in the study are deposited in the Genome Sequence Archive of the BIG Data Center at the Beijing Institue of Genomics, Chinese Academy of Science, under the accession number HRA001659 (HTTP://bigd.big.ac.cn/gsa-human). Code is available from corresponding author on resonable request.

## Ethics Statement

The studies involving human participants were reviewed and approved by the ethical committee at Shenzhen People’s Hospital (Reference number: LL-KY-2020199). The patients/participants provided their written informed consent to participate in this study.

## Author Contributions

Conceptualization: XZ and YH. Data acquisition: PK. Methodology: JZ, MZ, and LL. Data analysis: LL, FL, YX, XG, YG, XX, and XY. Writing original draft and editing: JZ, MZ, and YH. Data curation: JZ and YH. Project administration: XZ and YH. All authors read and approved the final manuscript.

## Conflict of Interest

Authors LL, FL, YX, XG, YG, XX, XY were employed by company Geneplus-Beijing.

The remaining authors declare that the research was conducted in the absence of any commercial or financial relationships that could be construed as a potential conflict of interest.

## Publisher’s Note

All claims expressed in this article are solely those of the authors and do not necessarily represent those of their affiliated organizations, or those of the publisher, the editors and the reviewers. Any product that may be evaluated in this article, or claim that may be made by its manufacturer, is not guaranteed or endorsed by the publisher.
