# Supplementary figures and images for: PIM1 and CD79B Mutation Status Impacts the Outcome of Primary Diffuse Large B-Cell Lymphoma of the CNS
Source: Front Oncol. 2022 Feb 9;12:824632. doi: 10.3389/fonc.2022.824632 (PMC8864287; doi:10.3389/fonc.2022.824632)

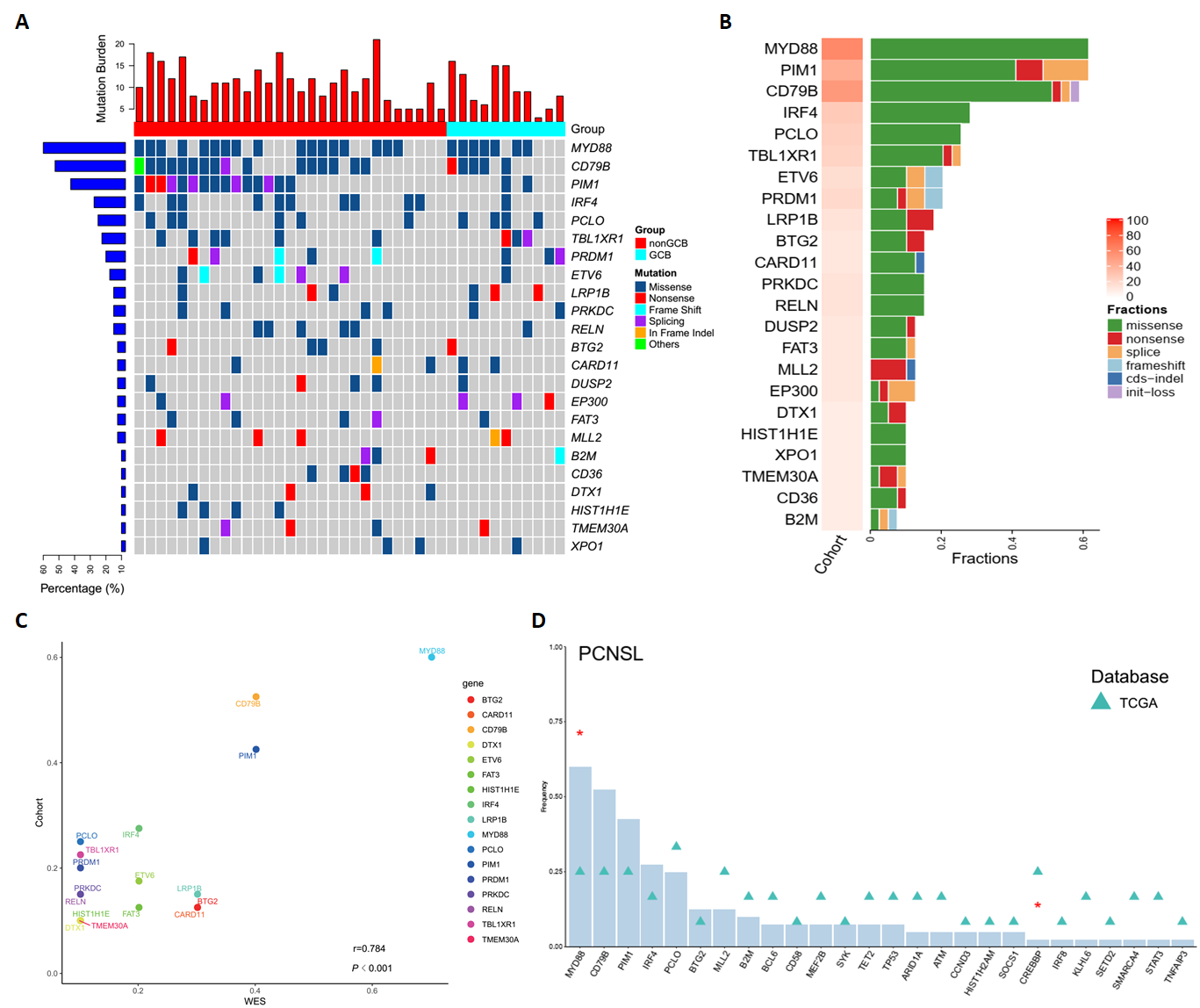

Supplement: Supplementary file 1 [file Image_1.tif]

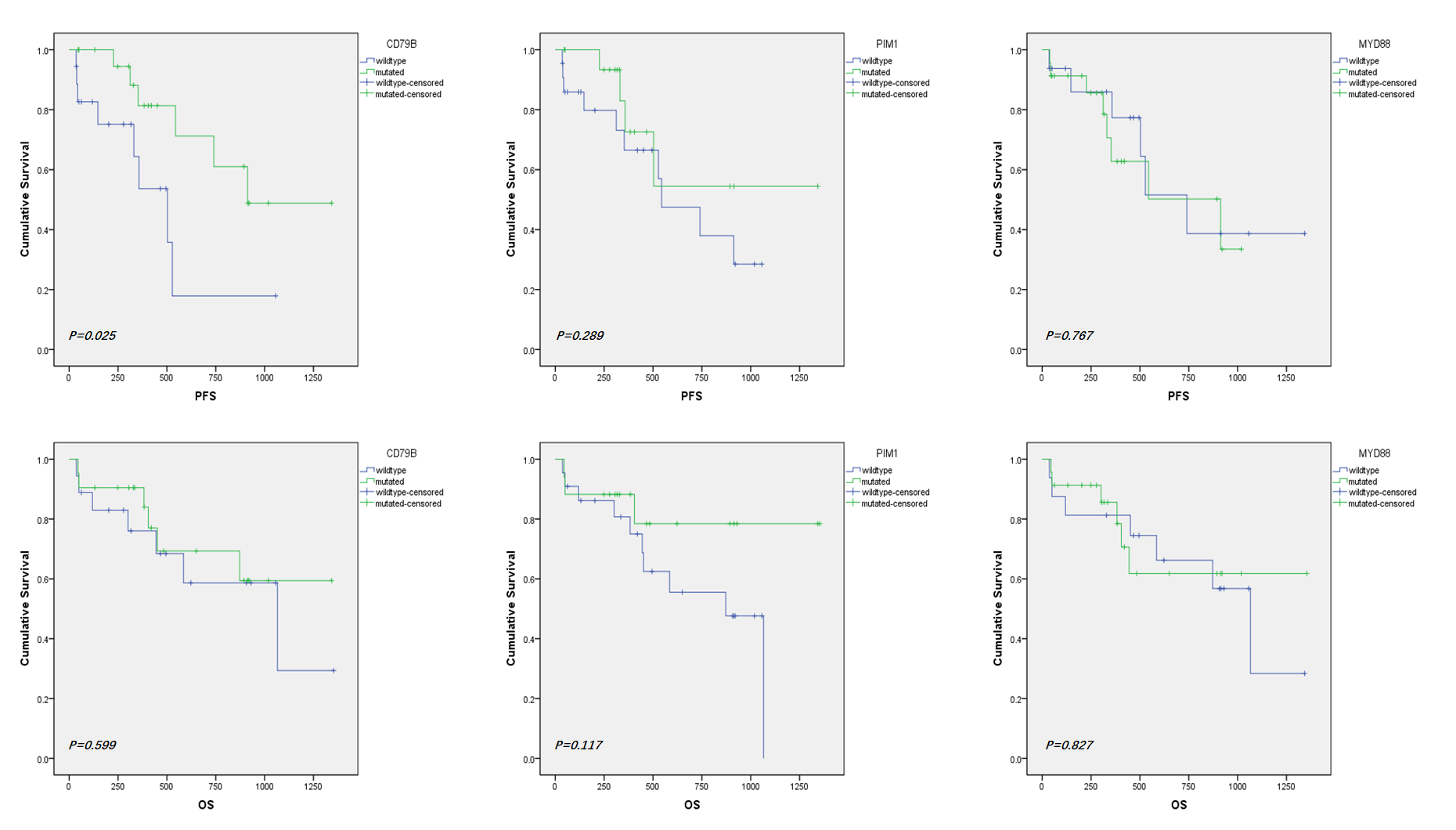

Supplement: Supplementary file 2 [file Image_2.tif]

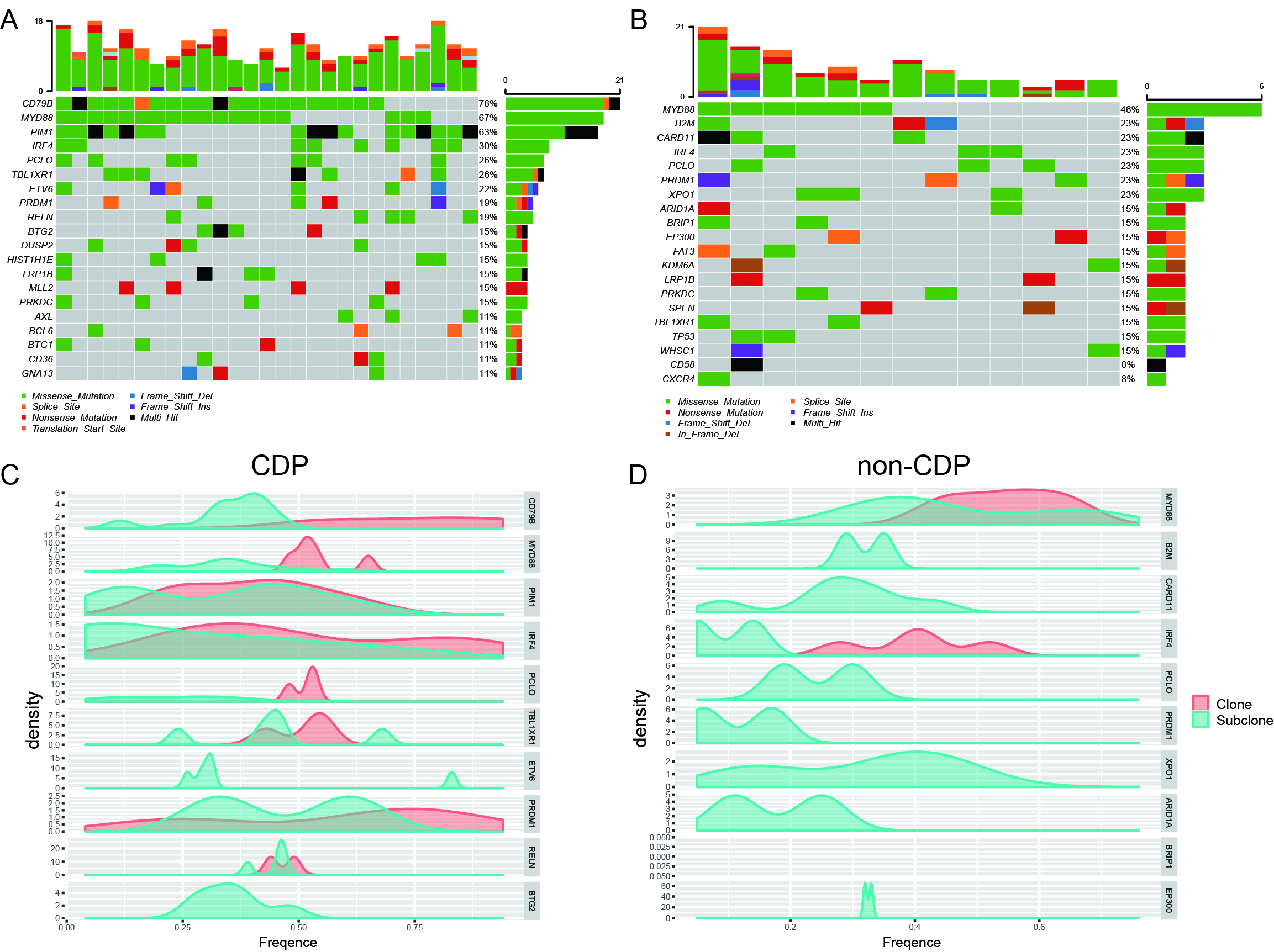

Supplement: Supplementary file 3 [file Image_3.tif]
